# Activation of the DR3-TL1A Axis in Donor Mice Leads to Regulatory T Cell Expansion and Activation With Reduction in Graft-Versus-Host Disease

**DOI:** 10.3389/fimmu.2019.01624

**Published:** 2019-07-17

**Authors:** Melissa Mavers, Federico Simonetta, Hidekazu Nishikii, Jessica V. Ribado, Kristina Maas-Bauer, Maite Alvarez, Toshihito Hirai, Mustafa Turkoz, Jeanette Baker, Robert S. Negrin

**Affiliations:** ^1^Division of Stem Cell Transplantation and Regenerative Medicine, Department of Pediatrics, Bass Center for Childhood Cancer and Blood Diseases, Stanford University School of Medicine, Palo Alto, CA, United States; ^2^Division of Blood and Marrow Transplantation, Department of Medicine, Stanford University Medical Center, Stanford, CA, United States; ^3^Department of Genetics, Stanford University School of Medicine, Stanford, CA, United States

**Keywords:** regulatory T cells, death receptor 3, TNFRSF25, TNF-like ligand 1A, TNFSF15, graft-versus-host disease

## Abstract

Death receptor 3 (DR3) is a tumor necrosis factor receptor superfamily member (TNFRSF25), which is minimally expressed on resting conventional T cells (though readily inducible upon cell activation), yet highly expressed on resting FoxP3^+^ regulatory T cells (Treg). We recently demonstrated that activation of DR3 with an agonistic antibody (4C12) leads to selective expansion and activation of Treg in healthy mice and suppression of graft-versus-host disease (GVHD) in recipient mice when donor mice are treated. However, given the long antibody half-life and concomitant safety concerns, along with the lack of a humanized agonistic antibody to DR3, both human and murine fusion proteins incorporating the natural DR3 ligand TL1A (TL1A-Ig) have been developed. Herein, we show that DR3 activation with 4C12 or with TL1A-Ig, with or without the addition of low dose IL-2 to the treatment regimen, led to a significant expansion of murine Treg in spleen, lymph nodes, and peripheral blood. Bioluminescent imaging revealed peak Treg expansion around day 7–8, with return to near baseline after 2–3 weeks. In addition to expansion, all DR3 agonist treatment regimens led to increased activation of Tregs, with significant upregulation of the activation markers ICOS, KLRG-1, PD-1, and CD103, and the proliferation marker Ki-67. The near absence of activated Treg populations in control treated spleens was also detected on tSNE analysis of flow cytometry data. Subtly different patterns of splenic Treg activation by the different DR3 agonists were noted in both tSNE analysis of flow cytometry data and RNA-sequencing analysis. However, upregulation of gene transcripts which play important roles in cell proliferation, trafficking, activation, and effector function were observed regardless of the DR3 agonist treatment regimen used. In the major MHC-mismatch model of hematopoietic cell transplantation, DR3 agonist-mediated expansion and activation of Tregs in donor mice led to a significant improvement in GVHD in recipient mice. These data provide important preclinical information regarding the outcome of DR3 activation with an agonistic antibody or natural ligand and provide insight into the therapeutic use of this approach to reduce GVHD in recipients and improve outcomes of hematopoietic cell transplantation.

## Introduction

Tumor necrosis factor (TNF) superfamily members, including ligands (TNFSF) and receptors (TNFRSF), play critical roles in influencing the immune landscape (1). Receptor ligation can enhance inflammation through the activation of nuclear factor kappa-light-chain-enhancer of activated B cells, mitogen-activated protein kinase signaling, and other signaling pathways in target cells (2). In particular, many of these ligand-receptor pairs, such as OX40-OX40L, 41BB-41BBL, and GITR-GITRL, have been shown to have a profound effect on T cell modulation including cell proliferation, reduction in apoptosis, and cytokine production (3).

The TNFRSF member death receptor 3 (DR3, TNFRSF25), is a type I transmembrane protein with homology to TNFR1 (4, 5). Expression of this receptor is highly inducible on T cells, and has also been detected on NKT cells (6) and innate lymphocytes (7, 8), as well as B cells and monocytes/macrophages (2). The natural ligand of DR3, TNF-like ligand 1A (TL1A, TNFSF15), is expressed on endothelial cells, antigen presenting cells, and T cells themselves (3). This receptor-ligand pair has been implicated in a variety of autoimmune diseases such as inflammatory bowel disease, rheumatoid arthritis, and psoriasis (9, 10).

While DR3 expression is rapidly induced on T cells following activation, constitutive expression on FoxP3^+^ regulatory T cells (Treg) has been demonstrated (11). We have previously shown that *in vivo* DR3 activation by an agonistic antibody (4C12) leads to significant expansion and activation of Treg (12, 13). While Treg play critical roles in many immune-mediated diseases, particular attention has been paid to the unique immune environment of allogeneic hematopoietic cell transplantation (HCT). HCT is curative for many high-risk malignancies and other disorders of blood and bone marrow. However, the use and efficacy of HCT is limited by the morbidity and mortality associated with graft-versus-host disease (GVHD), an allogeneic reaction of donor T cells to damaged host tissues (14, 15). Treg have been demonstrated to significantly reduce the severity of GVHD in both mouse models and humans (16–20), but clinical use is limited by difficulty in obtaining sufficient number of Treg either through direct isolation or expansion to clinically relevant numbers. We also previously investigated the effect of DR3-mediated Treg activation and expansion on GVHD and found that adoptive transfer of T cells from 4C12 treated mice significantly reduced GVHD in allogeneic recipients as compared to recipients of T cells from isotype control animals (12). Activation of this receptor has also been shown to protect against allergic lung inflammation (11) and improve cardiac allograft acceptance (21) through Treg effects. To further understand DR3 activation, and in particular the effect of its natural ligand, a fusion protein incorporating TL1A was generated (TL1A-Ig) (22). The TL1A domain of this fusion protein was found to form a trimer, as is characteristic of TNFSF members (23), and owing to the dimeric structure of the Ig domain results in a hexameric fusion protein (22).

In this study, we demonstrate the extent of expansion, activation phenotype, and suppressive function of Tregs exposed to DR3 activation by each agonist (4C12 or TL1A-Ig) as well as the effect of the addition of low dose IL-2. Our data show that activation of DR3 by any agonist treatment regimen leads to significant Treg expansion and activation resulting in suppression of GVHD, though subtle differences in the activation profiles were noted. These observations provide additional insight into the effects of these DR3 agonists, which is critical in understanding how to adapt these strategies for clinical translation.

## Materials and Methods

### Mice

Wild-type C57BL/6 (H-2k^b^ CD45.2^+^) and Balb/c (H-2k^d^ CD45.2^+^) mice were purchased from Jackson Laboratory. *Luc*^+^ C57BL/6 (B6-*luc*, H-2k^b^ Thy-1.1^+^ CD45.1^+^) mice were bred in our animal facility at Stanford University. FoxP3.Luci.DTR-4 mice (B6-albino background with the expression of luc/gfp proteins under control of the *foxp3* promoter) were a kind gift from Günter Hämmerling (German Cancer Research Center, Heidelberg, Germany) (24). Mice were used between the ages of 8–15 weeks. All animal protocols were approved by the Institutional Animal Care and Use Committee at Stanford University.

### Antibodies and Reagents

The FcR-blocking reagent, magnetic microbeads, and LS columns were purchased from Miltenyi Biotec (Auburn, CA). Agonistic anti-DR3 mAb (clone: 4C12) and Hamster IgG isotype control mAb (clone: HTK888) were purchased from Biolegend (San Diego, CA). Murine TL1A-Ig was kindly provided by Pelican Therapeutics/Heat Biologics (Durham, NC). Recombinant human IL-2 was a kind gift from the BRB pre-clinical repository of the NCI/NIH. Antibodies for use in flow cytometry were purchased from Biolegend or eBioscience (San Diego, CA), as listed in Supplemental Table 1. Fixable Viability Dye eFluor 506 (eBioscience) was used to exclude dead cells. Fixation/Permeabilization concentrate/diluent and permeabilization buffer were purchased from eBioscience.

### DR3 Activation, Cell Isolation, and Analysis

Mice were injected intraperitoneally (IP) with 4C12 (0.5 mg/kg) or isotype control (0.5 mg/kg) on day 1, or with TL1A-Ig (50 μg/dose) on days 1–4. Certain groups also received IP injections of low dose IL-2 (1 × 10^6^ units/m^2^ ≅ 3.3 × 10^5^ units/kg) on days 4 and 6. Tissues were harvested on day 7 and single-cell suspensions were prepared for analysis or use in the major mismatch GVHD model. Flow cytometric data were acquired on an LSR II instrument (BD Biosciences, San Jose, CA) and analyses were performed using FlowJo software (TreeStar, Ashland OR). Analyses included standard two-dimensional gating as well as t-distributed stochastic neighbor embedding (tSNE) analysis, which allows mapping of high-dimensional data (such as the 12 markers in our panel) onto two dimensions while preserving the high-dimensional structure (25). For RNA-sequencing (RNA-Seq), Treg were sorted based on GFP expression and RNA was extracted using Trizol (ThermoFisher, Waltham, MA) method. mRNA was purified with the KAPA mRNA HyperPrep Kits (KAPA Biosystems/Roche, Pleasanton, CA) and cDNA libraries were prepared using IDT for Illumina Dual Index Adapter kit per manufacturer instructions at the Stanford Functional Genomics Facility. Equal concentrations of cDNA library from each sample were pooled for sequencing on the Illumina HiSeq 4000 platform (75 bp, paired-end). Samples were sequenced at a median depth of 73,105,772 (range 50,886,214–105,048,450) reads. Sequencing reads were checked for adaptor contamination and quality using FastQC v. 0.11.8 with Q30 >90% for all reads per sample. Paired-end reads were aligned to the mm10 genome (GRCm38.p6) using STAR v. 2.5.4, and genes were quantified using the ‘–quantMode GeneCounts' option. The datasets generated for this study can be found in Sequence Read Archive, accession: PRJNA529746, https://www.ncbi.nlm.nih.gov/sra/PRJNA529746. Analyses were performed using R version 3.5.1. Differential gene expression was performed using the DESeq2 version 1.22.2 with pairwise contrasts (26). Differentially expressed genes were visualized using EnhancedVolcano version 1.1.3 and Pheatmap version 1.0.12. To identify the molecular-level activities and biological processes differentially regulated following DR3 agonist treatment, those genes with an adjusted *p*-value < 0.05 and up- or down-regulated at least 1.5-fold in DR3-agonist treated groups compared to isotype control were analyzed in the Enrichr analysis tool using Gene Ontology terms (2018) (27, 28). Enrichr uses the Benjamini-Hochberg (BH) procedure to correct for multiple hypotheses and provides the *p*-value (computed using Fisher's exact test), z-score (computed to assess the deviation from the expected rank), and combined score (combines the *p*-value and z-score calculated by: c = ln(p)^*^z where c is the combined score).

### Major MHC-Mismatch Model

Allogeneic HCT was performed as previously described (29). Briefly, female Balb/c recipient mice were irradiated with 880 cGy in 2 split doses on day 0. T cell-depleted bone marrow (TCD-BM) cells were isolated from wild-type non-treated sex-matched C57Bl/6 mice and donor T cells were isolated via magnetic bead separation (CD4 and CD8 beads, Miltenyi Biotec) of splenocytes harvested on day 7 from sex-matched C57BL/6 donor mice treated with one of the DR3 activation regimens described above. 5 × 10^6^ TCD-BM cells and 1 × 10^6^ splenic T cells were intravenously injected to recipients. Clinical evidence of GVHD was scored as previously described (30).

### Bioluminescence Imaging

*In vivo* bioluminescence imaging (BLI) was performed as previously described (31). Briefly, mice were injected with luciferin (10 mg/kg; IP), anesthetized, and imaged. Imaging was conducted using an IVIS Spectrum charge coupled device imaging system (Caliper-Xenogen, Alameda, CA) and data analysis with Living Image Software (Caliper Life Sciences, Hopkinton, MA) or using an Ami LED-illumination based imaging system (Spectral Instruments Imaging, Tucson, AZ) and data analysis with Aura Software (Spectral Instruments Imaging).

### Statistical Analyses

Flow data are displayed as mean and standard deviation and analyzed by one-way ANOVA with Bonferonni correction for multiple comparisons. BLI data are displayed as mean and standard error for each group over time and analyzed by two-way ANOVA with Bonferonni correction. Weight loss and GVHD clinical score are displayed as mean and standard error for each group over time and analyzed by multiple student *t*-tests with Holm-Sidak correction. Survival curves were plotted with the Kaplan-Meier method and compared by log-rank test. Statistical significance as compared to isotype control group is displayed for all figures. All statistical analyses were performed using Prism 6 (GraphPad Software, La Jolla, CA).

## Results

### Activation of DR3 Transiently Expands Regulatory T Cells

**Figure 1:**
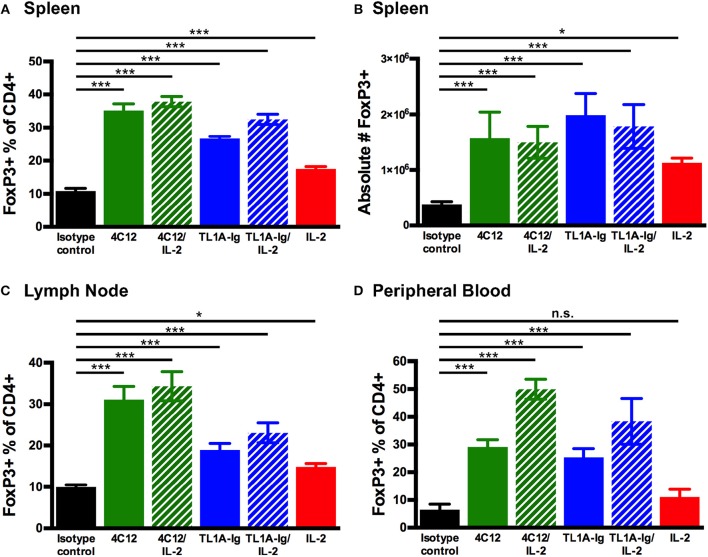
Regulatory T cells are expanded through *in vivo* activation of the death receptor 3 pathway with agonistic antibody (4C12) or fusion protein incorporating the endogenous ligand (TL1A-Ig) with or without IL-2. Mice were treated as in Materials and Methods. Tissues were harvested on day 7 of the treatment regimen, processed and stained, and analyzed by flow cytometry. Live cells were gated on the lymphocyte population, followed by CD3^+^CD19^−^, CD4^+^ and FoxP3^+^. Shown are the percent FoxP3^+^ of CD4^+^ cells in **(A)** splenocytes, **(C)** lymphocytes, and **(D)** peripheral blood. Also shown are the **(B)** absolute number of regulatory T cells in splenocytes. (Shown are the mean and SD for each group (*n* = 4). n.s., not significant; ^*^p < 0.05 and ^***^p < 0.001 as compared to isotype control antibody treatment, one-way ANOVA with Bonferroni correction for multiple comparisons). Data shown are representative of 2 independent experiments.

To demonstrate the effect of different strategies of DR3 activation on Treg expansion, mice were injected with DR3 agonistic antibody (4C12) or TL1A-Ig fusion protein (TL1A-Ig) with or without IL-2 as described in *Materials and Methods*. Tissues were harvested on day 7 of the treatment regimen and evaluated for Treg expansion by flow cytometry (Supplemental Figure 1). Treg were significantly increased following all treatment regimens as compared to isotype control antibody in spleen (Figures 1A,B), lymph nodes (Figure 1C), and peripheral blood (Figure 1D). Because invariant natural killer T (iNKT) cells are known to express DR3 (6), we also investigated whether this population was expanded following activation by 4C12 or TL1A-Ig, with or without IL-2. On day 7, the iNKT population was not significantly expanded in spleen, lymph nodes, or peripheral blood from mice treated with any DR3 agonist regimen (data not shown).

**Figure 2:**
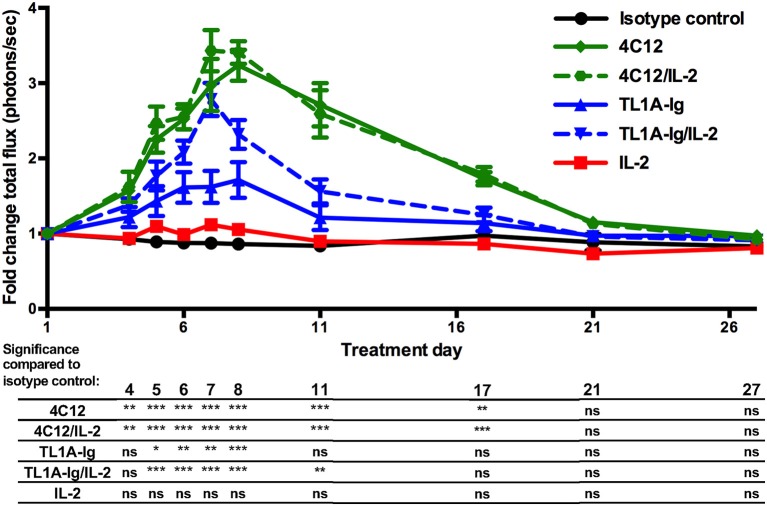
Kinetics of *in vivo* expansion of regulatory T cells through activation of the death receptor 3 pathway. Mice expressing luciferase under control of the FoxP3 promoter were treated with agonists of the DR3 pathway and regulatory T cell expansion was analyzed via bioluminescent imaging as in Materials and Methods. (Shown are the mean and SEM for each group (*n* = 8) over time. ^*^p < 0.05, ^**^p < 0.01, and ^***^p < 0.001 as compared to isotype control antibody treatment, two-way ANOVA with matched values and Bonferroni correction for multiple comparisons). Results are combined from 2 independent experiments.

Bioluminescent imaging using mice expressing luciferase under control of the FoxP3 promoter (C57BL/6-FoxP3.Luci.DTR-4) allowed for the visualization of the time course of DR3-mediated Treg expansion for each treatment group. We again demonstrated that DR3 activation using either agonist led to a significant increase in Treg signal as compared to isotype control antibody treated mice. Both 4C12 and TL1A-Ig treatment regimens lead to peak expansion around day 7–8 (Figure 2). The expansion was transient and returned to near baseline levels within 3–4 weeks for all groups.

### Regulatory T Cells Expanded by DR3 Agonists Display an Activated Phenotype

**Figure 3:**
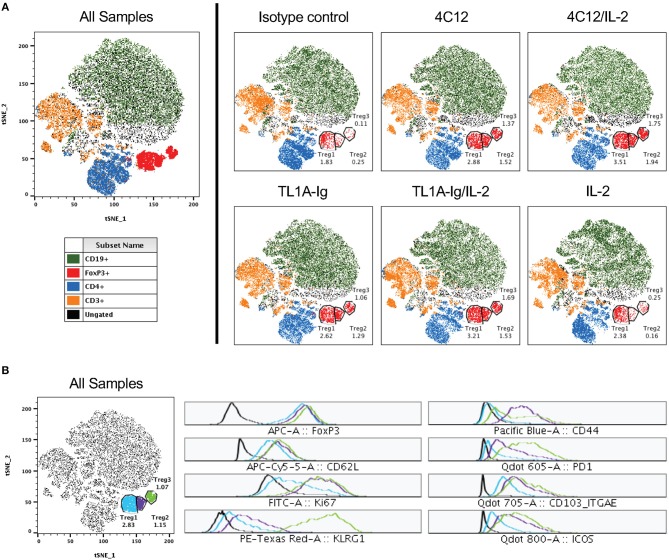
Regulatory T cells comprise a heterogenous population with distinct activated subsets significantly increased following DR3 activation. Live lymphocytes (gated on FSC versus SSC and a live/dead marker) from all samples were concatenated and subjected to tSNE analysis (data shown are pooled from two independent experiments). **(A)** Shown on left is the tSNE plot for all samples and on right for each treatment group. Clustered populations were identified using the indicated markers and three regulatory T cell populations are gated as shown. The percent of cells in each Treg population are shown for each treatment group on the tSNE plots. **(B)** Displayed on left is the tSNE plot for all samples from **(A)** with each Treg population differentially labeled. At right, histograms for each Treg population are shown for the indicated analytes.

We utilized the t-Distributed Stochastic Neighbor Embedding (tSNE) dimensionality reduction algorithm to map our high-dimensional flow cytometry data (including 12 markers of phenotype and activation status) onto two dimensions without loss of the hierarchical structure (25). This analysis on concatenated splenic lymphocytes from mice in all treatment groups revealed that the Treg population was sufficiently heterogenous to cluster into three populations (Figure 3A). When viewing each treatment group separately using this tSNE map, we found that one population (Treg1) was only slightly increased (≤ 2-fold) in the DR3-agonist treated groups as compared to control groups (isotype antibody or IL-2). However, two of the populations (Treg2 and Treg3) were nearly absent in the control-treated mice, but drastically increased in those mice undergoing DR3 activation (about 5- to 10-fold increase). These two Treg populations represent highly proliferative and activated Tregs, including upregulation of Ki67, KLRG-1, PD-1, CD103, and ICOS, while the population well-represented in all treatment groups including controls (Treg1) displays minimal to no activation (Figure 3B).

**Figure 4:**
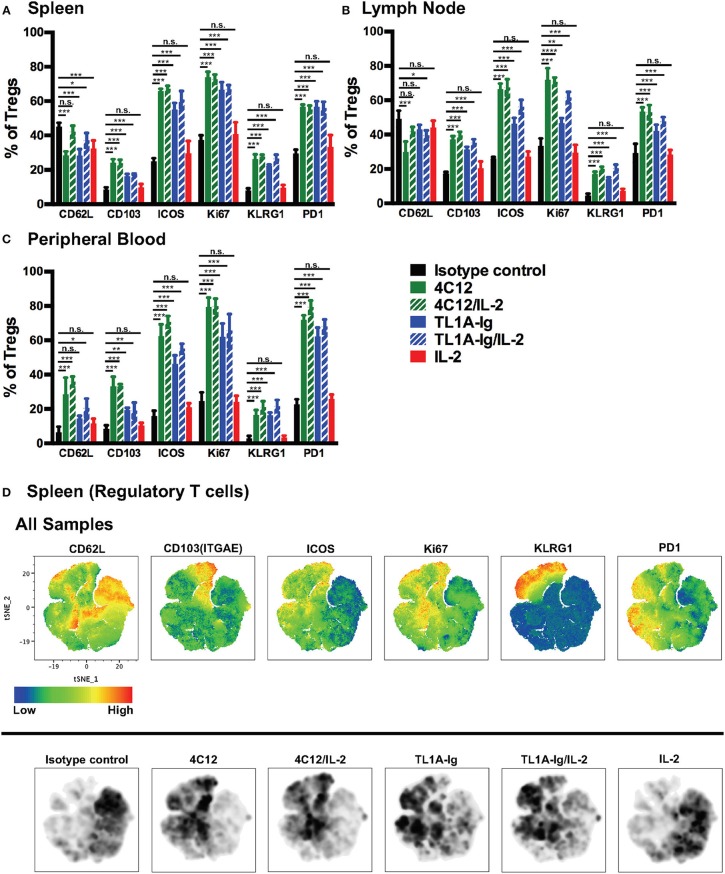
*In vivo* stimulation of death receptor 3 leads to activation of expanding regulatory T cells. Tissues were isolated from mice treated as in Materials and Methods on day 7 of the treatment regimen, single cell suspensions prepared, and cells were analyzed by flow cytometry. Shown are the expression levels of activation makers in FoxP3^+^ regulatory T cells from **(A)** spleen, **(B)** lymph nodes, and **(C)** peripheral blood. (Shown are the mean and SD for each group (*n* = 4). n.s., not significant; ^*^p < 0.05, ^**^p < 0.01, and ^***^p < 0.001 as compared to isotype control antibody treatment, one-way ANOVA with Bonferroni correction for multiple comparisons). Data shown are representative of 2 independent experiments. **(D)** Splenic FoxP3^+^ Treg were gated from live lymphocytes, concatenated from all samples, and subjected to tSNE analysis. Upper panels show heat maps of all concatenated samples for the indicated analytes. Lower panels show density plots for each treatment group (*n* = 4) on the same tSNE map, with darker areas indicating more cells present in that region.

To view this DR3 agonist-mediated activation on a more granular level for each marker in each treatment group, we performed standard two-dimensional flow cytometry analyses. Significant upregulation of ICOS, KLRG-1, PD-1, CD103, and Ki67, but not CD62L, was noted on splenic Tregs from mice treated with DR3 agonists as compared to isotype control (Figure 4A). Similar results were seen on Treg from lymph nodes (Figure 4B) and peripheral blood (Figure 4C), although CD62L was increased on peripheral blood-derived Tregs from mice treated with DR3 agonists relative to isotype control. We then performed a tSNE analysis on splenic Treg concatenated from all treatment groups and determined which regions of the tSNE map had the highest expression levels of each marker (Figure 4D, upper panels). We then used density plots to determine where the majority of the Treg from each treatment group fell on the same tSNE map, which allowed us to see qualitative differences between the groups (Figure 4D, lower panels). Interestingly, treatment with 4C12 versus TL1A-Ig lead to differential activation (or expansion) of Treg in that Treg more densely populated the region highly expressing CD103 following treatment with 4C12 (+/-IL-2) whereas Treg were more dense in the area highly expressing PD-1 following treatment with TL1A-Ig (+/-IL-2).

### RNA-Sequencing Reveals Upregulation of Transcripts of Genes Involved in Cell Proliferation and Immune Activation in DR3-agonist Treated Regulatory T Cells

**Figure 5:**
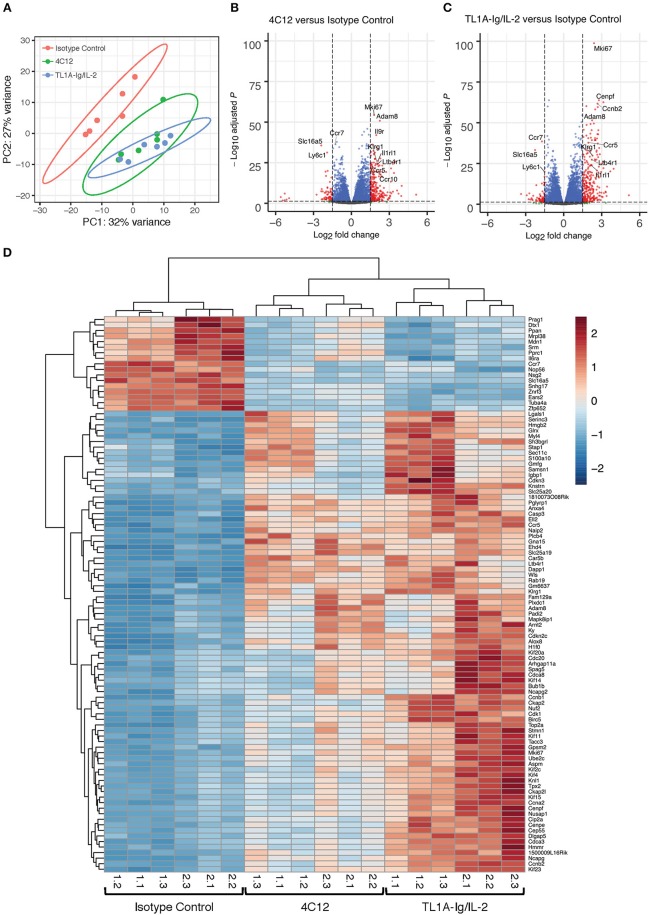
RNA-Sequencing reveals upregulation of transcripts involved in cell proliferation and immune activation in DR3-agonist treated Tregs. Mice expressing GFP under control of the FoxP3 promoter were treated with isotype control antibody, DR3 agonistic antibody (4C12), or fusion protein incorporating the endogenous DR3 ligand (TL1A-Ig) with IL-2 as in Materials and Methods. Splenic Treg were isolated by sorting on GFP and RNA-sequencing was performed. **(A)** Principle component analysis reveals clustering of DR3 agonist treated groups away from isotype control. **(B,C)** Volcano plots showing significance and Log2 fold change of transcripts from DR3 agonist treated groups compared to isotype control reveal enrichment for genes involved in proliferation, trafficking, activation, and effector function. Vertical dashed lines on volcano plots indicate a Log2 fold change of 1.5; horizontal dashed line indicates an adjusted *p*-value of 0.05. **(D)** Heatmap displaying the 100 most highly differentially expressed genes. Column labels below the heatmap give “experiment #.replicate #” for each treatment group. Data shown are pooled from two independent experiments.

To further define the effect of DR3 activation on Treg, bulk RNA-seq was performed on splenic Treg from mice treated with isotype control antibody, 4C12, or TL1A-Ig/IL-2. Unsupervised principal component analysis demonstrated a clustering of isotype control samples away from DR3-agonist treated samples, while there was significant overlap in the samples from 4C12 and TL1A-Ig/IL-2 treated groups (Figure 5A). Transcripts were plotted by adjusted *p*-value versus fold change for each treatment group as compared to isotype control (Figures 5B,C) and the 100 most highly differentially expressed genes were shown in a heatmap, in which unsupervised hierarchical clustering clearly distinguished DR3-agonist treated groups from isotype control, although a batch effect was noted between experiments (Figure 5D). Unsurprisingly, given the significant proliferation of Tregs following DR3 activation, many of the highly upregulated genes are involved in cellular replication processes. In addition, several genes were highly upregulated in DR3 agonist treated Treg which play important roles in their immunological function, such as trafficking, cytokine-mediated activation, and effector function/phenotype stability. Gene Ontology analysis similarly revealed activation of these pathways (Supplemental Tables 2–9). The upregulation of Leukotriene B4 Receptor 1 (*Ltb4r1*) and members of the C-C chemokine receptor family (*Ccr5* and *Ccr10*) following DR3 activation suggests an increased propensity for trafficking to target tissues, as does the increase in Adam8, involved in extravasation from blood vessels (32). Similarly, the relative downregulation of CCR7 following DR3-activation (enriched in isotype control treated Treg) suggests cells are primed to egress from lymphoid tissue and traffic to non-lymphoid target tissues (33). Upregulation of the receptor for interleukin (IL)-9 (*Il9r*) in DR3-agonist treated Treg suggests increased responsiveness to this cytokine, which has been shown to enhance the suppressive capacity of Treg (34). Interestingly, IL-1 receptor like 1 (*Il1rl1*, also known as IL-33 receptor, or ST2) was also upregulated following DR3-agonist treatment. Not only have ST2^+^ Treg been shown to exhibit an activated phenotype (35), but ST2^−/−^ donor Treg have a reduced capacity to suppress GVHD (36). In addition, IL-33 sequestration from the membrane-bound ST2 receptor has been shown to play a pathogenic role in GVHD and adversely affect Treg numbers (36). Importantly, the significant upregulation of killer cell lectin-like receptor subfamily G member 1 (*Klrg1*) supports our flow cytometry data and suggests enhanced effector function (37). Taken together, the DR3 agonist-mediated upregulation of a number of genes involved in proliferation, trafficking, activation, and effector function of Treg in our RNA-seq data, combined with our flow cytometry data revealing similar effects at the protein level, and our previously reported results, prompted us to investigate the use of DR3 agonists in suppression of GVHD.

### Donor Treatment With 4C12 or TL1A-Ig With or Without IL-2 Leads to Reduced GVHD Morbidity and Mortality

**Figure 6:**
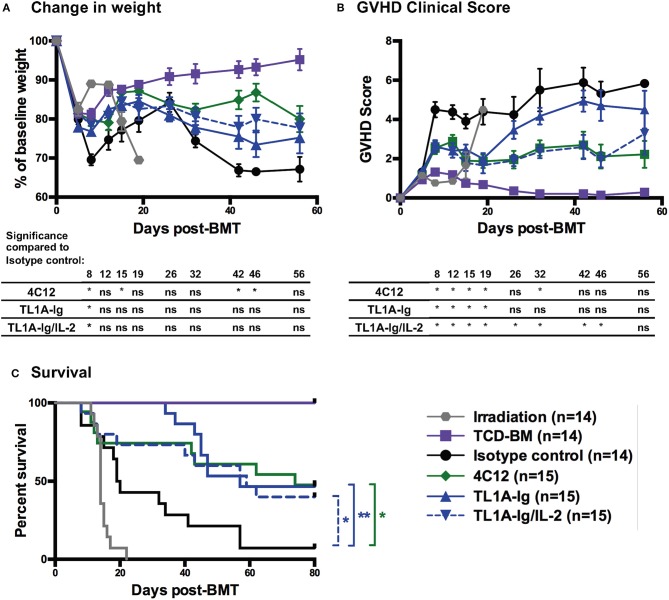
Donor treatment with DR3 agonistic antibody (4C12) or fusion protein incorporating the endogenous ligand (TL1A-Ig) with or without IL-2 leads to a reduction in acute GVHD severity. T cells were isolated from donor mice (C57Bl/6) treated with DR3 agonists on day 7 of the treatment regimen and injected into Balb/c recipients along with T cell depleted bone marrow as in Materials and Methods. GVHD was assessed by measurement of **(A)** weight loss, **(B)** GVHD clinical score, and **(C)** survival. (Shown are the mean and SEM for each group over time. ^*^p < 0.05 and ^**^p < 0.01 as compared to isotype control, **(A,B)** multiple student *t*-tests with Holm-Sidak correction and **(C)** log-rank test). Results are combined from 3 independent experiments. TCD-BM, T-cell depleted bone marrow only control.

The effect of DR3-mediated Treg expansion and activation in donor mice on the development of acute GVHD in recipients was examined using a major MHC-mismatch model of acute GVHD. Donor mice were treated according to the regimens noted in *Materials and Methods*. Splenic T cells were harvested on day 7 of the treatment regimen and used in the GVHD model. Disease severity was monitored through assessment of change in weight, GVHD clinical score, and survival (Figure 6). While weight loss was not significantly improved at most time points evaluated (Figure 6A), GVHD clinical score was improved in recipients of splenocytes from mice in all DR3-activated treatment groups throughout the initial wave of GVHD (and throughout the entire evaluation period for recipients of T cells from mice in the TL1A-Ig/IL-2 treatment group) (Figure 6B). Donor treatment with the DR3 agonistic antibody (4C12) or the TL1A-Ig fusion protein with or without IL-2 also led to a survival benefit in recipients as compared to those receiving T cells from donors treated with isotype control antibody (Figure 6C).

## Discussion

Better understanding the nuances of TNF superfamily receptor-ligand interactions will allow for more specific tailoring of therapeutics designed to block these interactions (such as when and how to use TNF-blocking antibodies in autoimmune disease) and the ability to exploit these interactions when enhanced immunity is desired (as in cancer immunotherapy). Indeed, further study of TNFRSF members may reveal unanticipated effects, such as the enhancement of Treg number and function following activation of DR3 as demonstrated previously and in this report (12, 13, 38, 39).

We have shown that activation of DR3 with an agonistic antibody (4C12) in healthy mice leads to a robust and selective expansion of Treg (12, 13), likely due to constitutive DR3 expression on Treg with minimal to no expression on resting conventional T cells (11). In this report, we have again demonstrated this action of 4C12, as well as that of a fusion protein incorporating the natural DR3 ligand TL1A-Ig (22). Despite reported constitutive expression of DR3 on invariant natural killer T cells (6), no expansion of this population was noted on day 7 following receptor activation. While DR3 ligation may lead to different kinetics of iNKT cell expansion or activation without expansion, these data further highlight the possibility of divergent effects of the same receptor on different cell populations.

In addition to Treg expansion, both 4C12 and TL1A-Ig treatment led to significant upregulation of activation markers, including ICOS, KLRG-1, PD-1, and CD103, detected by flow cytometry. However, subtly different patterns of activation by the different DR3 agonists were noted both on tSNE analysis of flow data and in RNA-Seq analysis. Despite this, Treg from both 4C12 and TL1A-Ig/IL-2 treatment regimens clustered away from Treg treated with isotype control antibody in principal component analysis of RNA-Seq data. The majority of genes upregulated following DR3 activation with either treatment regimen play important roles in cell proliferation, which correlated with the high levels of Ki67 observed by flow cytometry. Genes involved in Treg trafficking, activation, and effector function were significantly upregulated as well. Notably, Ly6c1 was enriched in isotype control treated Treg. This supports previously reported data in which the Ly6c1^+^ (but not Ly6c1^−^) central Treg subset is drastically reduced in spleens from DR3-agonist treated mice (38, 39).

Treg have been previously shown to play a critical role in suppression of GVHD (both for prevention and treatment) in several different murine models as well as in humans (16–19, 31, 40–43). In this report, we show that DR3-mediated expansion and activation of Treg in donor mice led to enhanced function in the suppression of GVHD. In particular, there was an improvement in GVHD clinical scores and a survival benefit regardless of the agonist used. Although the addition of low dose IL-2 to the TL1A-Ig donor treatment regimen appeared to prolong the improvement in GVHD clinical score in recipient mice, both regimens were effective in reducing GVHD mortality.

These studies provide important preclinical data demonstrating the ability of a fusion protein incorporating the TNFSF member TL1A to expand and activate regulatory T cells resulting in suppression of GVHD. Because of the lack of a human DR3 agonistic antibody, the translational potential of results showing the efficacy of 4C12 in Treg expansion is limited. Our previous work has shown that 4C12-mediated DR3 activation in HCT *recipients* led to variable effect on GVHD depending on timing of the dose (13). In those recipients treated with 4C12 prior to the adoptive transfer of donor T cells to induce GVHD, there was a protective benefit to the treatment. However, when recipients were treated at the same time as adoptive transfer of GVHD-inducing T cells, GVHD was significantly worsened. This was primarily due to upregulation of the DR3 receptor on conventional T cells activated by exposure to alloantigens. Therefore, it is apparent that for clinical translation of this approach, donor treatment will be required—further highlighting the benefit of using a fusion protein (with a shorter half-life and likely better safety profile) rather than an agonistic antibody. Importantly, the immunosuppressive role for the DR3-TL1A axis that we have demonstrated may have critical therapeutic implications not only for stem cell transplantation, but also for many other clinical scenarios such as autoimmune disease. In addition, these findings underscore the need for further study of this and all members of the TNFRSF to better understand their nuanced functions.

## Data Availability

The datasets generated for this study can be found in Sequence Read Archive, accession: PRJNA529746, https://www.ncbi.nlm.nih.gov/sra/PRJNA529746.

## Ethics Statement

This study was carried out in accordance with the recommendations of the USDA Animal Welfare Act and the PHS Policy on Humane Care and Use of Laboratory Animals. The protocol was approved by the Stanford University Administrative Panel on Laboratory Animal Care (APLAC), which is accredited by the Association for the Assessment and Accreditation of Laboratory Animal Care (AAALAC International).

## Author Contributions

MM, HN, and RN contributed to the conception and design of the study. MM, FS, KM-B, MA, TH, MT, and JB contributed to collection of data. MM, JR, and FS contributed to analysis of data. MM, FS, and RN contributed to interpretation of results. MM wrote the first draft of the manuscript, with significant input from RN. MM, FS, and JR wrote sections of the manuscript. All authors contributed to manuscript revision, read, and approved the submitted version.

### Conflict of Interest Statement

The authors declare that the research was conducted in the absence of any commercial or financial relationships that could be construed as a potential conflict of interest.
