# Supplementary figures and images for: Activation of the DR3-TL1A Axis in Donor Mice Leads to Regulatory T Cell Expansion and Activation With Reduction in Graft-Versus-Host Disease
Source: Front Immunol. 2019 Jul 17;10:1624. doi: 10.3389/fimmu.2019.01624 (PMC6652149; doi:10.3389/fimmu.2019.01624)

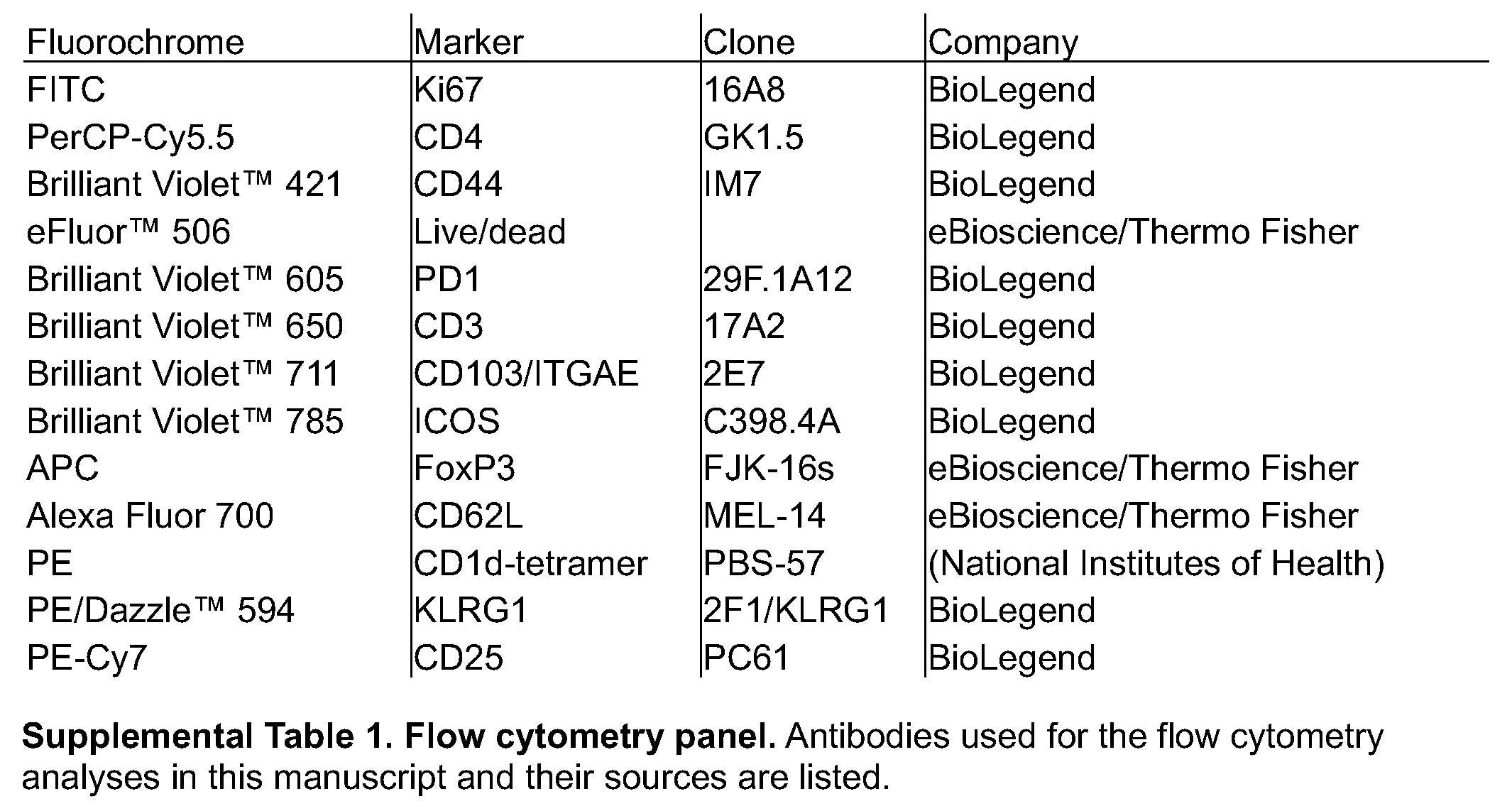

Supplement: Supplementary file 1 [file Table_1.docx]

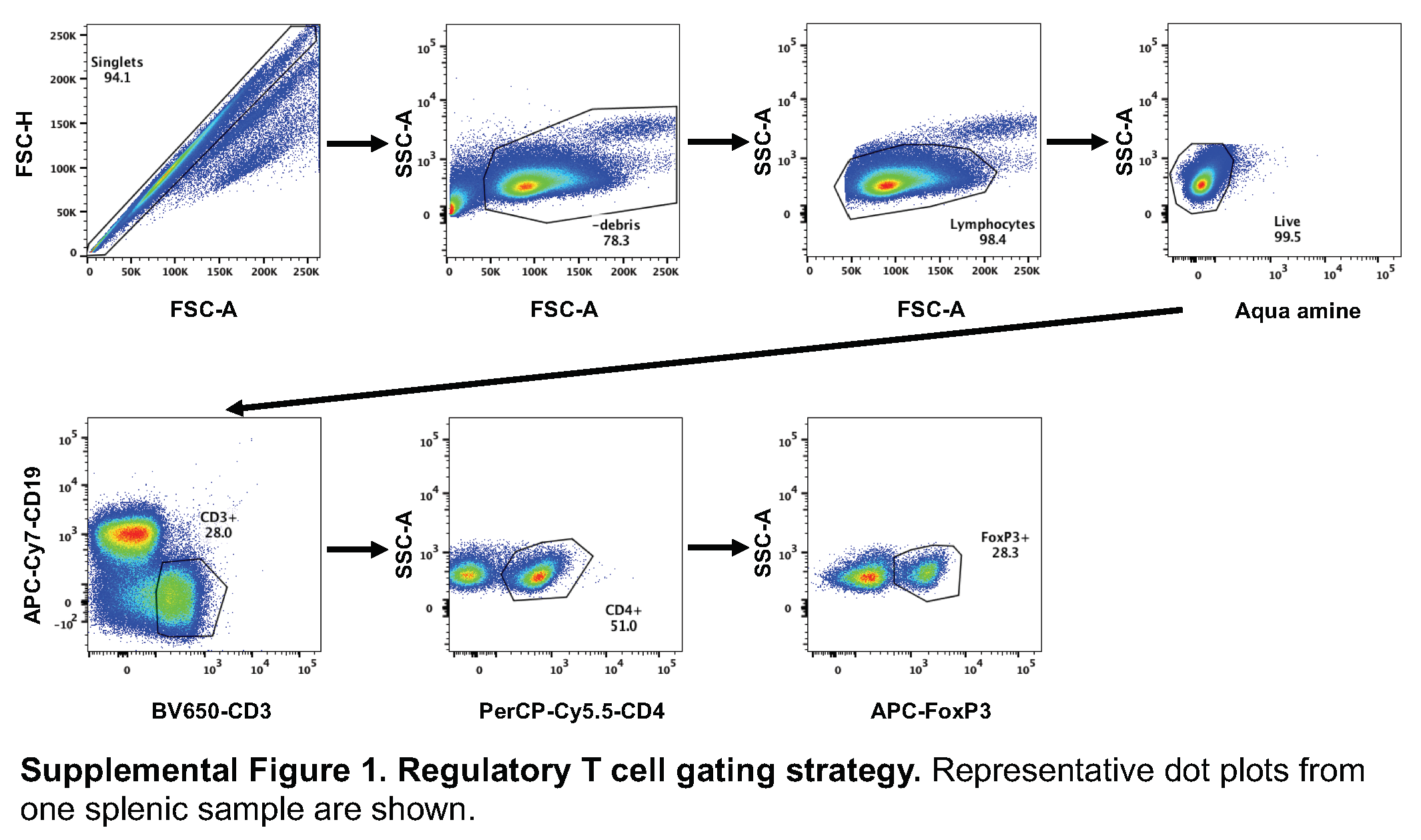

Supplement: Supplementary file 3 [file Image_1.TIFF]
